# Late infection of thoracic aortic stent graft with aerodigestive fistula: a case series and narrative literature review

**DOI:** 10.3389/fcvm.2026.1819955

**Published:** 2026-05-29

**Authors:** Chang Jinxing, Zheng Yao, Min Xinping, Xia Jun, Xu Peng, Wu Hongbing, Hu Xiaoping, Wu Qi

**Affiliations:** Department of Cardiovascular Surgery, Renmin Hospital of Wuhan University, Wuhan, Hubei, China

**Keywords:** aortic stent graft infection, aorto-esophageal fistula, aorto-tracheal fistula, case series, outcome

## Abstract

**Objective:**

To describe the clinical presentation, diagnostic process, and surgical outcomes of late thoracic aortic stent graft infection (ASGI) with aerodigestive fistula.

**Methods:**

We retrospectively analyzed three late ASGI patients admitted between 2015 and 2025, supplemented by a narrative literature review. Case 1 presented with mediastinal abscess and aorto-esophageal fistula; Case 2 with aorto-esophageal and aorto-tracheal fistula; Case 3 with aorto-tracheoesophageal fistula.

**Results:**

All three patients underwent surgical intervention: abscess debridement and drainage (Case 1); re-intervention with covered stent, esophageal resection, and lobectomy (Case 2); and staged aortic bypass (Case 3). The 30-day postoperative mortality rate was 1/3 (33.3%), and the 1-year mortality rate was 3/3 (100%).

**Conclusion:**

Late ASGI complicated by aerodigestive fistula carries an extremely poor prognosis. Early recognition and timely consideration of definitive source control may be important, but optimal management remains challenging.

## Introduction

Infection after aortic stent graft implantation is a catastrophic complication in vascular surgery, with an incidence of approximately 0.5%–3%, but a mortality rate as high as 25%–75% (1). With the surge in TEVAR/EVAR procedures, the diagnostic and therapeutic challenges of ASGI in the medium to long term (>4 weeks) have become increasingly prominent (2). Unlike early infections, late ASGI often manifests as chest or back pain or peri-graft fluid accumulation, with severe cases presenting as aorto-tracheo-esophageal fistula, and delayed diagnosis is common.

At present, there is still controversy over the treatment of ASGI in international guidelines (3). This study reports three typical long-term ASGI cases, with a time span of 3–9 years after stent implantation. These cases demonstrate three invasion modes: aorto-esophageal fistula with mediastinal abscess (Case 1), aorto-esophageal and aorto-tracheal fistula (Case 2), and aorto-tracheoesophageal fistula (Case 3). This article aims to provide empirical evidence for clinical decision-making through multiple case analysis.

## Case report

### Case 1

**Figure 1:**
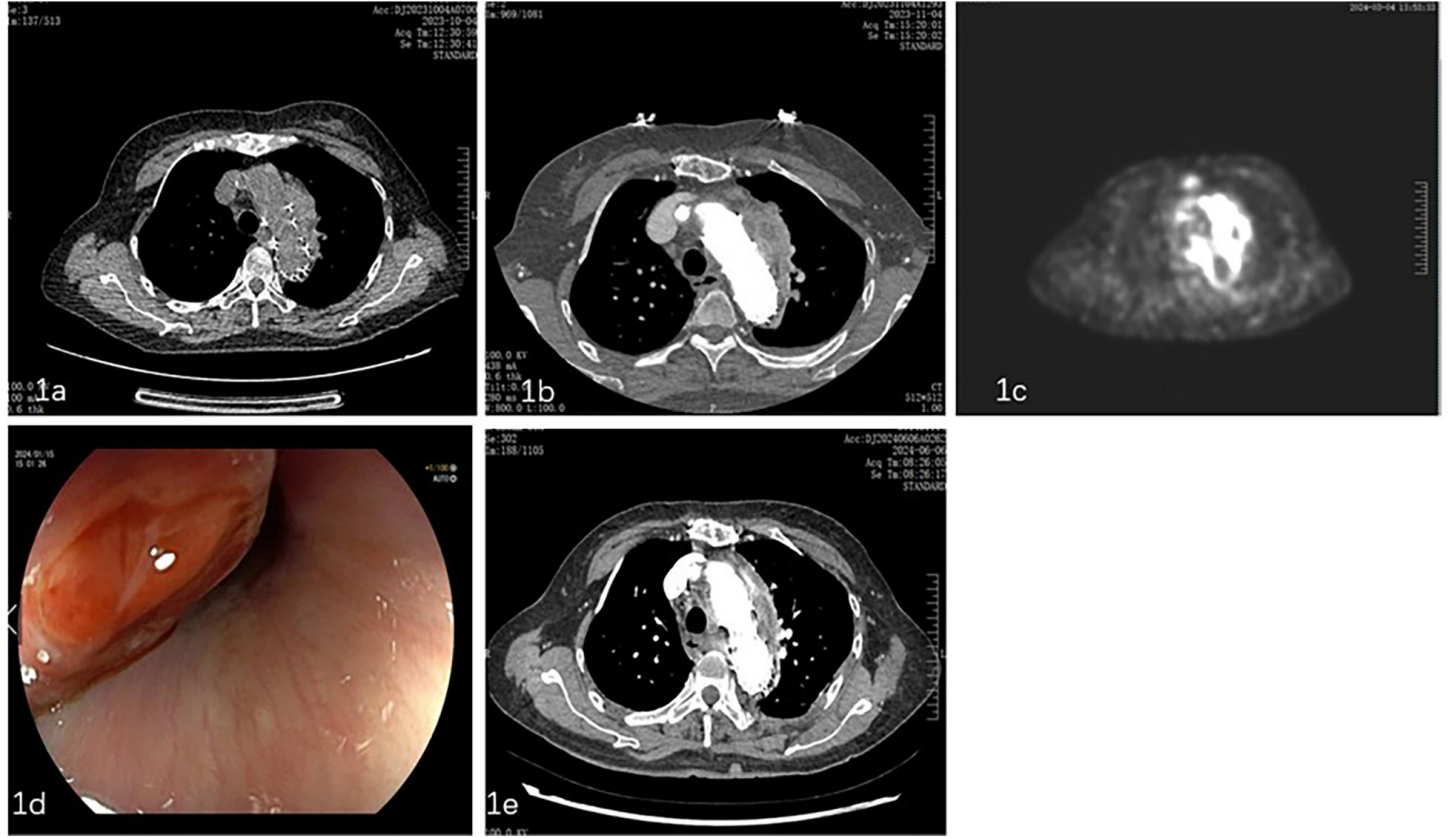
**(a,b)** chief complaint of chest and back pain with dysphagia, CT suggests mediastinal abscess. **(c)** 18F-FDG PET-CT suggests a lobulated soft tissue mass in the left upper mediastinum with increased metabolism, and localized increased metabolism in the middle esophagus, suggesting a possible infectious lesion. **(d)** Gastroscopy suggests external compression of the upper esophagus. **(e)** Postoperative CT scan showed a significant reduction in mediastinal foci.

A 68-year-old female underwent Sun's surgery for Stanford Type A aortic dissection in January 2015. She has a history of hypertension and percutaneous coronary intervention (PCI) for the anterior descending branch of the coronary artery in 2014 due to coronary heart disease. Since October 2023, the patient has complained of “chest and back pain” and visited the outpatient department multiple times, undergoing chest CT or chest pain triple examination (Figures 1a,b). Three months later, dysphagia with pain after eating occurred. Gastroscopy suggested external compression of the upper esophagus (Figure 1d). On February 29, 2024, the patient was admitted for treatment of mediastinal abscess. A whole-body 18F-FDG PET-CT scan (Figure 1c) showed a soft tissue mass in the left upper mediastinum with increased metabolism, and localized increased metabolism in the middle esophagus, suggesting a possible infectious lesion. One month later, the patient underwent a mediastinal abscess debridement and drainage procedure, and was discharged smoothly three weeks later. On June 4, the patient was readmitted due to ‘hematemesis'. Angiography did not reveal any obvious bleeding site, and the patient was given blood transfusion and other treatments. However, the patient died from massive hematemesis again one week later. No further surgical intervention was performed between discharge and readmission.

### Case 2

**Figure 2:**
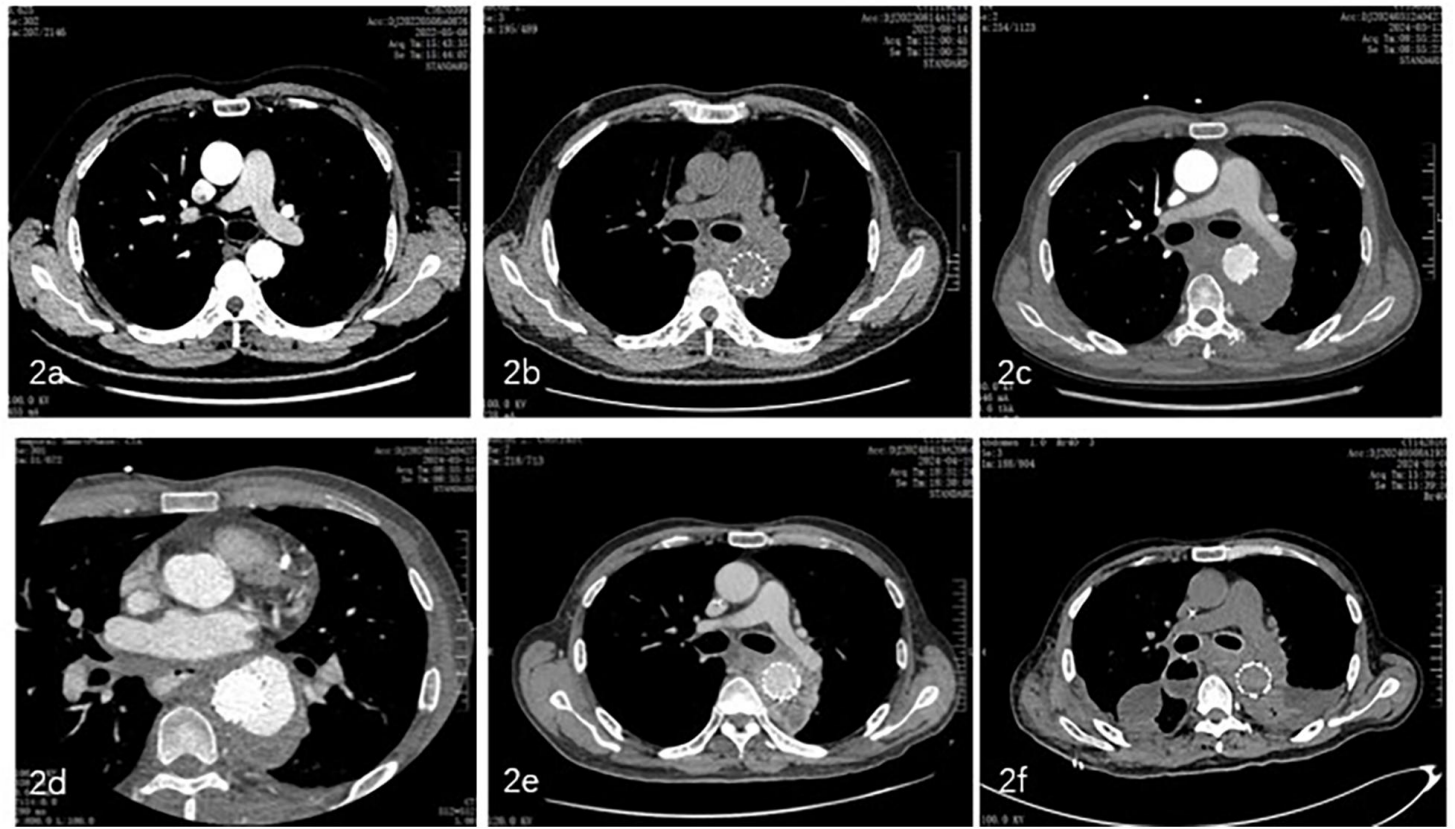
**(a)** chest CT scan six months after TEVAR. **(b)** Two years after TEVAR, fluid accumulation around the stent graft; **(c,d)** Two and a half years after TEVAR, the patient was found to have increased fluid accumulation accompanied by contrast extravasation on a CT scan during a follow-up for hematemesis. **(e)** Follow-up aortic CTA after re-implantation of a covered stent shows persistent fluid around the stent. **(f)** Chest CT scan for postoperative hemoptysis after undergoing gastric substitution for esophagus via the right thoracic cavity due to hematemesis.

A 55-year-old male patient was hospitalized in August 2021 due to two days of chest pain. Coronary angiography showed severe stenosis of the left anterior descending artery, prompting percutaneous coronary intervention (PCI). Concurrently, a triple-rule-out test for chest pain indicated a penetrating ulcer in the descending segment of the aortic arch, accompanied by intramural hematoma formation. Two months later, he underwent thoracic endovascular aortic repair (TEVAR) (with a follow-up at six months post-surgery, as shown in Figure 2a). In May 2023, he was repeatedly admitted due to “chest discomfort with shortness of breath” (with a follow-up at two years post-surgery, as shown in Figure 2b), and received symptomatic treatment. By March 2024, he presented with hematemesis, and gastroscopy revealed a protruding lesion in the mid-esophagus. Aortic computed tomography angiography (CTA) raised the suspicion of a thoracic aortic-esophageal fistula (as shown in Figures 2c,d). TEVAR was performed at the distal end of the stent (as shown in Figure 2e), leading to an improvement in hematemesis and stabilization of his general condition. On April 28, 2024, he underwent a partial esophagectomy with gastric substitution for the esophagus and a nutritional jejunostomy through a right thoracotomy involving cervical, thoracic, and abdominal incisions. Two weeks later, he underwent a left lower lobe resection due to hemoptysis (as shown in Figure 2f). Intraoperative findings revealed inflammatory granulation tissue around the aorta. The patient ultimately succumbed to multi-organ failure secondary to infection two weeks after the bleeding episode.

### Case 3

**Figure 3:**
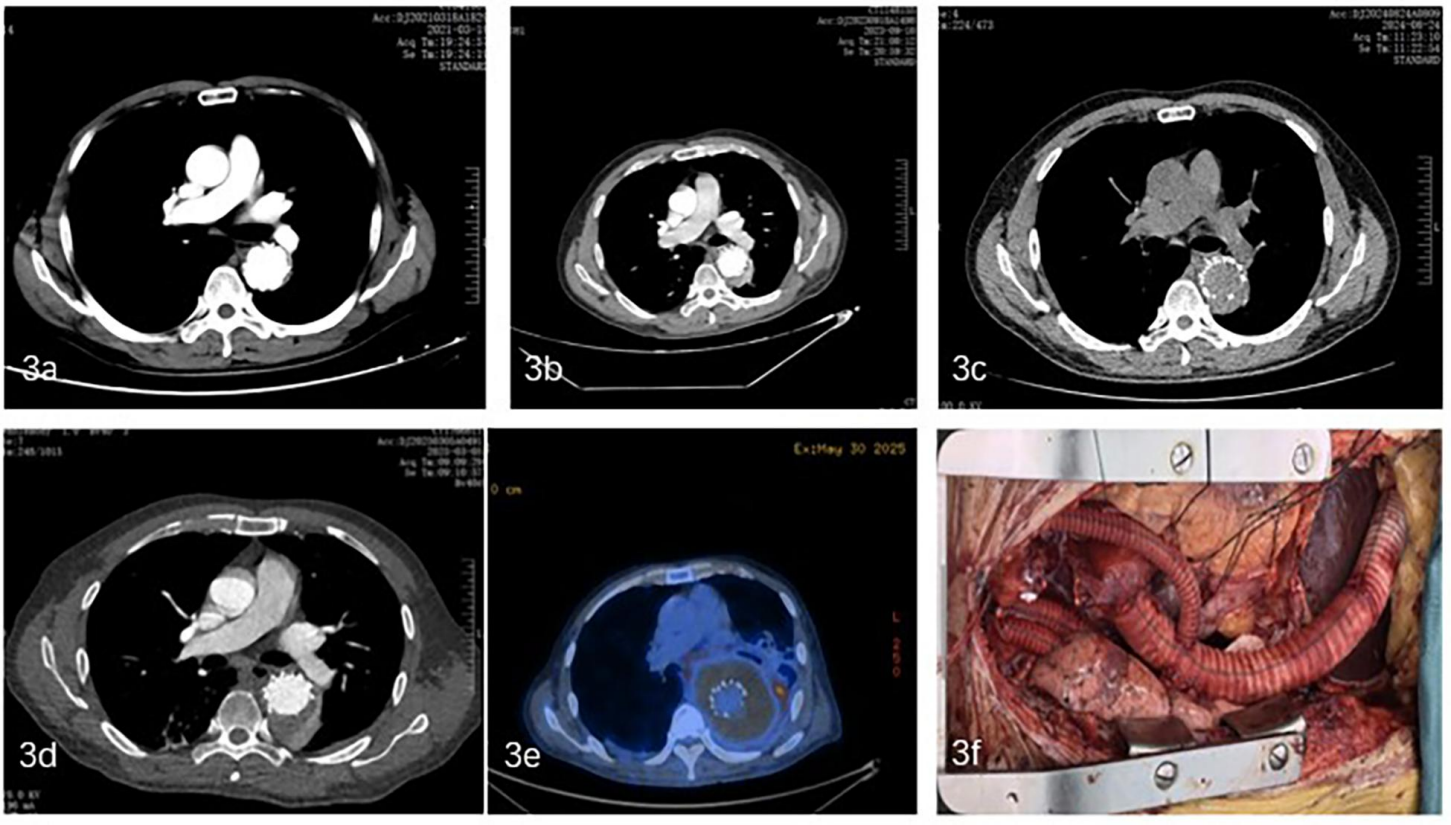
**(a)** three-year follow-up CTA after TEVAR. **(b,c)** Fluid accumulation around the stent 5–6 years after TEVAR. **(d)** Seven years after TEVAR, there is a significant increase in fluid accumulation around the stent graft. Seven years after TEVAR, there is a significant increase in fluid accumulation around the stent graft. **(e)** Seven years after TEVAR, the patient developed hemoptysis. **(f)** The first stage surgery involves ascending aorta-abdominal aorta artificial vascular bypass and reconstruction of the three branches of the aortic arch.

A 57-year-old male patient underwent TEVAR (Figure 3a) due to aortic dissection in 2018. Since February 2023, he has repeatedly experienced discomfort characterized by “chest pain accompanied by chest tightness” and sought outpatient treatment (Figures 3b,c). In August 2024, he was admitted to the hospital for “chest pain” and underwent coronary angiography (CAG), which showed no significant stenosis. From February to March 2025, he was hospitalized multiple times for “abdominal pain” (Figure 3d). Gastroscopy revealed reflux esophagitis and erosive gastritis. Symptomatic and supportive treatment was provided, leading to an improvement in his symptoms. In mid-May 2025, he was hospitalized again due to “high fever”. After admission, he developed hemoptysis. An 18F-FDG PET-CT scan indicated an aortic stent infection (Figure 3e). Following anti-infective and hemostatic treatments, his symptoms improved. On June 1st, he underwent a one-stage ascending aorta-to-abdominal aorta bypass grafting under general anesthesia (Figure 3f). The infected stent was scheduled to be removed in a second-stage procedure. However, two weeks after the first-stage surgery, he suddenly suffered from uncontrollable hematemesis and died. The diagnostic workup, microbiology results, and clinical timelines for the three patients are summarized in Tables 1, 2, and 3, respectively.

**Table 1:** Diagnostic criteria (MAGIC) and workup for each patient.

Criterion	Case 1	Case 2	Case 3
Peri-graft fluid ≥3 months	Yes	Yes	Yes
Aorto-esophageal/tracheal fistula	Yes	Yes	Yes
Fever (>38 °C)	No	No	Yes
Leukocytosis (>12,000/*μ*L)	No	No	Yes
Positive blood culture	No	No	No
Positive PET-CT	Yes	No	Yes
MAGIC diagnosis	Confirmed	Confirmed	Confirmed

**Table 2:** Microbiology and antibiotic therapy.

Category	Case 1	Case 2	Case 3
Blood culture	Negative	Negative	Negative
Intraoperative culture	Negative	Negative	Not done
Empirical antibiotics	Piperacillin/tazobactam	Meropenem	Vancomycin+Meropenem
Definitive therapy	None	None	None (no pathogen identified)

**Table 3:** Timeline of key events for each patient.

Event	Case 1	Case 2	Case 3
Index aortic procedure	Jan 2015 (Sun's surgery)	Oct 2021 (TEVAR)	2018 (TEVAR)
First symptom (pain)	Oct 2023	May 2023	Feb 2023
First peri-graft fluid on CT	Oct 2023	May 2023	Feb 2023
Fistula diagnosed	Feb–Mar 2024	Mar 2024	May 2025
Surgical intervention	Mar 2024 (drainage)	Apr 2024 (esophagectomy)	Jun 2025 (bypass)
Death	Jun 2024	May 2024	Jun 2025

## Discussion

Vascular grafts (including grafts placed via open surgery and endovascular stent-grafts implanted using minimally invasive techniques) are increasingly used to treat life-threatening aortic aneurysms, aortic dissections, and obstructive vascular diseases. The incidence of aortic graft infection is approximately 0.5% to 4% (2). The basic principles of treatment include removal of the infected graft, vascular reconstruction, and antibiotic therapy. In infected cases, the surgical mortality rate can be as high as 18% to 30% (4, 5). For conservative treatment, the mortality rate within 2 years can approach 100% (6, 7). ASGI is highly complex in diagnosis and treatment, with diverse clinical manifestations, and there is currently no ‘gold standard' of diagnostic principles, also there is a lack of existing relevant clinical studies such as radiology, microbiology, and drug therapy (5, 8). ASGI is generally diagnosed through the integrated results of clinical, radiological, and laboratory findings, but the diagnostic criteria lack precision. Currently, the diagnosis of Active Graft Infection mainly refers to the diagnostic criteria proposed by the Aortic Graft Infection Management Collaboration Group (MAGIC) in 2013 (9). Suspected ASGI is indicated when a patient presents with any single major criterion from clinical/surgical, imaging, or laboratory categories, or two minor criteria from these three categories. A diagnosis of active graft infection can be made when there is a single major criterion, along with any other criterion (major or minor) from another category. In Case 1, peri-graft fluid was visible on CT several months before fistula formation, but the absence of fever and negative inflammatory markers delayed the diagnosis of late ASGI. This highlights the need for a high index of suspicion when peri-graft fluid persists more than 3 months after implantation, even in afebrile patients. Regarding the use of PET-CT when CT and endoscopy already suggested graft infection, PET-CT was performed in Cases 1 and 3 to assess the extent of infection and rule out other occult foci. However, we agree that its added diagnostic value was limited in these cases. PET-CT may be most useful when CT findings are equivocal.

Late-stage stent infection presents with different clinical symptoms from early infection, which may be without fever and have insufficient microbiological evidence (10). The time span from symptom onset to complication occurrence, such as fistula formation, is long (11). In these 3 cases, the time span from the frequent occurrence of thoracolumbar pain or abdominal pain after stent implantation to the development of aortic stent tracheal or esophageal fistula (hemoptysis or hematemesis) was approximately 8–24 months. Moreover, once thoracolumbar pain symptoms became frequent, chest CT scans showed a significant increase in fluid accumulation around the stent, and regular chest CT examinations are helpful in determining whether stent infection should be considered (12). However, the low incidence of ASGI and the diversity of clinical symptoms increase the difficulty of diagnosis. Among the reported cases, two patients underwent esophagogastroduodenoscopy (EGD) due to gastrointestinal symptoms. Both examinations revealed exophytic lesions in the upper or middle esophagus. Therefore, for patients suspected of having late-onset infection of thoracic aortic stent grafts, endoscopic examination is warranted when the clinical condition permits, as it aids in disease diagnosis and selection of surgical approach.

Observations made by the European Vascular Surgery Prosthetic Research Group (GEPROVAS) through examination of explanted aortic grafts and endografts indicate that both stent fabric and structural degradation are present, and these are more severe in the inflammatory environment caused by infection, suggesting that graft degradation may interact with the occurrence of infection (13). A pathological study of arterial prostheses surgically excised after overt clinical infection revealed that the infection was found on the external capsule of the grafts rather than on the luminal surface (14). In Case 1, a mediastinal abscess was cleared during the operation, and postoperative CT scan showed a significant reduction in mediastinal foci (Figure 1e), perioperative cultures of blood and secretions were all negative. However, a mediastinal esophageal fistula leading to hematemesis occurred during postoperative follow-up. For Patient 2, although the diseased esophagus was resected, the untreated aortic lesion eventually involved the trachea, leading to an aorto-tracheal fistula. This indicates that, inflammatory lesions of the aorta itself rather than the graft are the root cause of severe consequences in late ASGI. Although these two factors are closely intertwined, their underlying mechanisms require further investigation.

Infections of aortic grafts at different anatomical sites present distinct challenges for surgical treatment (15). When facing such conditions, surgeons often exercise great caution in selecting the treatment approach, as the in-hospital mortality of both patients treated with endograft preservation and endograft ex-plantation reached a very high level (16). In this case series, only mediastinal abscess debridement and drainage were performed for Case 1, the postoperative CT showed apparent improvement, but the patient's hematemesis caused by the esophageal compression lesion identified during preoperative gastroscopy indicated that the mediastinal inflammation persisted and further deteriorated. For Case 2, due to uncontrollable hematemesis, an esophageal lesion incision and gastric replacement for the esophagus were performed via a right thoracic approach. During a lobectomy for postoperative hemoptysis, intraoperative findings revealed that the tissue surrounding the infected stent had necrotized and invaded the bronchus. For Case 3, given the patient's poor physical condition, a staged approach was planned: first, artificial vascular bypass surgery, and then followed by removal of the infected focus after general condition improvement. However, the patient suddenly developed hematemesis and ultimately had an adverse outcome.

The treatment strategy in each case was individualized based on patient condition, fistula type, and institutional experience. In Case 1, only debridement and drainage were performed due to the patient's frailty and high operative risk, despite guideline recommendations for definitive explantation and esophageal repair. In Case 2, re-stenting and esophageal resection were chosen to control life-threatening hematemesis, but the untreated aortic lesion later involved the trachea. In Case 3, a staged approach was planned because of poor general condition, but the patient died before the second stage. These cases illustrate that while definitive source control is the goal, it is often not feasible in unstable patients.

Based on our limited experience, the long-term prognosis of late ASGI with aerodigestive fistula is extremely poor. Definitive source control is challenging, especially in unstable patients. Early recognition of warning signs (persistent chest/back pain, peri-graft fluid on CT) and timely consideration of surgical intervention may be important, but optimal management remains uncertain and requires further study.

## Data Availability

The raw data supporting the conclusions of this article will be made available by the authors, without undue reservation.
